# Preface Biological Toxins—Ancient Molecules Posing a Current Threat

**DOI:** 10.3390/toxins7124888

**Published:** 2015-12-08

**Authors:** Brigitte G. Dorner, Andreas Rummel

**Affiliations:** 1 Biological Toxins, Centre for Biological Threats and Special Pathogens, Robert Koch Institute, 13353 Berlin, Germany; 2 Institut für Toxikologie, Medizinische Hochschule Hannover, 30623 Hannover, Germany

Based on their characteristics, biological toxins are at the interface of classical biological and chemical agents: for example, ricin and saxitoxin are prohibited substances under both the Chemical Weapons Convention and the Biological Weapons Convention. The latter also prohibits bacterial toxins such as botulinum neurotoxins (BoNT) and staphylococcal enterotoxins (SE). Generally, biological toxins are substances produced by living organisms (e.g., plants, bacteria, algae), but they are not living, hence unable to reproduce, propagate or spread from person to person. Therefore, they share many of the characteristics of classical chemical agents (e.g., sarin). However, since high molecular weight proteins like ricin or BoNT exert an enzymatic function within the body amplifying their potency, they are clearly different from the low molecular-weight chemical warfare agents and, additionally, they display orders of magnitude of higher specific toxicity to humans.

Generally, biological toxins are relevant in the health and food sector as well as in the security sector. On the one hand, biological toxins are linked with natural intoxications worldwide and some of them cause severe and recurrent diseases (e.g., food poisoning induced by SE and marine toxins like saxitoxin; the life-threatening disease botulism induced by BoNT). On the other hand, the relative ease in preparing some of the mentioned toxins and the world-wide availability of the biological sources constitute them as potential agents of bioterrorism. The ricin-containing letters sent to U.S. President Obama and several other decision makers in the USA in 2003 and 2013 showed that biological toxins can be produced by laypersons and that they can be intentionally released in a criminal act to harm individuals or groups of people. In recognizing that biological toxins are a source of natural intoxications as well as potential agents of bioterrorism, it is important to know how well prepared our vulnerable, open societies are with respect to detection of biological toxins of potential bioterrorism risk. 

In this context, the European Union funded a project abbreviated EQuATox (“Establishment of Quality Assurances for the Detection of Biological Toxins of Potential Bioterrorism Risk”, grant agreement No. 285120, www.equatox.eu) from January 2012 to December 2014. One major goal of the project was to set up a network of nationally appointed laboratories for the detection of biological toxins of potential bioterrorism risk. The EQuATox project focused in-depth on biological toxins and their intricacies with respect to detection, identification, structure and function—topics that might become relevant for an efficient management of natural or man-made incidents involving biological toxins. The main task of EQuATox was to define the status quo of detection technology for biological toxins in EU-28 and associated countries. 

EQuATox gathered expert laboratories from the security, verification, health and food sector—35 laboratories from 20 countries worldwide—to work on biological toxins of potential bioterrorism risk, practising an efficient civilian-military cooperation. By building a network of expert laboratories and strengthening their technical exchange, the project already helped to improve the analytical capabilities within European countries in the neglected area of biological toxins. On a technical level, EQuATox addressed open questions with respect to detection, identification and quantification of both high and low molecular weight toxins. The project obtained highly important results with respect to reference materials, standardized detection and best practices in four large proficiency tests focusing on ricin, saxitoxin, staphylococcal enterotoxin B (SEB) and BoNT. The results are highlighted in a series of 13 articles in the current Special Issue “Detection and Identification of Biological Toxins in International Proficiency Tests” in *Toxins*.

From a diagnostic point of view, biological toxins are a challenging group of agents as they may act in the absence of the producing organism and its genetic information. Therefore, the focus of detection cannot be—as in the case of viruses and bacteria—on nucleic acid-based methods. Rather, the toxin itself has to be detected either by spectrometric, immunological, chromatographic and functional assays or combinations thereof. The precise detection and identification of biological toxins is difficult, since they naturally occur in different isoforms or variants: ricin, for example, occurs in the plant *Ricinus communis* in the isoforms ricin D and ricin E, accompanied by the 90% identical agglutinin. BoNT is released by the bacterium *Clostridium botulinum* and other *Clostridia* in seven different serotypes and can be grouped into more than 40 subtypes, which can significantly differ in their amino acid sequence. A similar variety occurs in SE with more than 26 isoforms.

Generally, different technologies for toxin detection and analysis are in use in expert laboratories. However, there are hardly any universally agreed “gold standard” methods, nor qualified or certified reference materials available for most of the toxins in focus (ricin, SE, BoNT). Expert laboratories currently use various purified in-house materials, which makes any comparison of accuracy and sensitivity of different methods nearly impossible. Furthermore, no interlaboratory exercises for the mentioned toxins had been performed, especially exercises which take into account detection from complex environmental matrices, clinical matrices or food. 

One major aim of EQuATox in facing these technical challenges was to identify good analytical strategies and to highlight any critical gaps in detection technology. Based on the solid information obtained in four large international proficiency tests—one on each of the above mentioned toxins—it is important to state that there is room for improvement in the standardisation level for the detection of biological toxins. The situation is clearly different to the field of bacterial and viral pathogens, where steps towards standardisation have been successfully taken on an international level. 

The aim of this *Toxins* Special Issue is to highlight the current status of toxin detection focusing on ricin, saxitoxin, SEB and BoNT. For each of the four toxins, a series of manuscripts describes the reference materials produced in the project, as well as the results of dedicated international proficiency tests taking into account the balance between information sharing and confidential issues (e.g., dual-use issues). Additionally, for each toxin different articles provide information on good analytical practices identified in the exercises. In this context, the data presented provides a solid basis and a starting point for future advancement in quality assurance efforts.

We would like to thank all authors contributing to this Special Issue in *Toxins* for their expert input throughout the project. Special thanks go to all proficiency test participants—it was their scientific input and experimental effort that allowed us to critically evaluate the *status quo* of toxin detection and to draw meaningful conclusions for future actions.

